# Role of SIRT3 in Angiotensin II-induced human umbilical vein endothelial cells dysfunction

**DOI:** 10.1186/s12872-015-0075-4

**Published:** 2015-07-30

**Authors:** Hui Liu, Tongshuai Chen, Na Li, Shujian Wang, Peili Bu

**Affiliations:** The Key Laboratory of Cardiovascular Remodeling and Function Research, Chinese Ministry of Education and Chinese Ministry of Public Health, Qilu Hospital, Shandong University, Jinan, 250012 Shandong Province China; Department of Cardiology, Qilu Hospital, Shandong University, No. 107 Wen Hua Xi Road, Jinan, 250012 Shandong Province China

**Keywords:** SIRT3, Endothelial dysfunction, AngII, Reactive oxygen species, HUVECs

## Abstract

**Background:**

SIRT3, a member of the sirtuin family of NAD^+^-dependent deacetylases, resides primarily in the mitochondria and has been shown to deacetylate several metabolic and respiratory enzymes that regulate important mitochondrial functions. Previous researches show an important role of SIRT3 in regulating the production of reactive oxygen species (ROS), and highlight the ability of SIRT3 to protect cells from oxidative damage. A key substance of renin-angiotensin-aldosterone system (RAAS), Angiotensin II (AngII) can induce cells dysfunction by increasing the production of ROS. In this paper, we focus on the role of SIRT3 in AngII-induced human umbilical vein endothelial cells (HUVECs) dysfunction.

**Methods:**

To study the influence of AngII on SIRT3 expression, HUVECs were treated with AngII of 10^−7^, 10^−6^, 10^−5^ mol/L for 24 h. SIRT3 expression was detected by wester-blotting analysis and RT-PCR. In addition, to research the role of SIRT3 in AngII-induced HUVECs,we used SIRT3 siRNA to knock down SIRT3 expression in HUVECs. Cells pretreated with negative control siRNA or SIRT3 siRNA were exposed to AngII for 24 h, and endothelial nitric oxide synthase (eNOS) expression, eNOS activity, total level of nitric oxide (NO) and ROS generation of each group were detected.

**Results:**

Here we show that AngII treatment could increase generation of ROS, and decrease eNOS activity and total level of NO, while upregulated eNOS expression as a compensatory mechanism. The stimulation of AngII upregulated the expression of SIRT3 in HUVECs. SIRT3 siRNA worsen the AngII-induced effects above, besides, downregulated eNOS protein expression.

**Conclusion:**

These data suggest that SIRT3 plays a role of protection in AngII-induced HUVECs dysfunction via regulation of ROS generation.

## Background

As the inner layer of the blood vessel wall, the endothelium plays the major role in the regulation of vascular homeostasis and is the target for a variety of neurotransmitters, hormones, or physiologic stimuli. Endothelial dysfunction, mainly refer to an impairment of endothelium-dependent vasorelaxation caused by a loss of nitric oxide (NO) bioactivity in the vessel wall, is observed in the presence of major cardiovascular risk factors, including atherosclerosis, hypertension, and heart failure [1–3]. A large body of evidence invariably demonstrates that the presence of endothelial dysfunction is a hallmark of the hypertensive patient [4]. Notably, endothelial dysfunction can occur at an early stage of hypertension, and may make an important contribution to the increase of blood pressure. On the other hand, endothelial dysfunction is considered as a consequence of hypertension, and, in these conditions endothelial dysfunction may contribute to further increases in peripheral vascular resistance and cardiovascular complications [5]. Reduced bioavailability of NO and abundant formation of reactive oxygen species (ROS) within the vascular wall are the key determinants in endothelial dysfunction [6].

Angiotensin II (AngII), a key substance of renin-angiotensin-aldosterone system (RAAS), can cause vasoconstriction, sympathetic nervous stimulation, release of aldosterone, and renal actions which contribute to control the blood pressure [7]. AngII can induce endothelial dysfunction by increasing the production of ROS through regulating the activity of nicotinamide adenine dinucleotide phosphate oxidase (NOX) [8]. Several studies have demonstrated that hypertensive patients and various animal models of hypertension produce excessive amount of ROS, and have abnormal levels of antioxidant status, thereby contributing to the accumulating evidence that increased vascular oxidative stress could be involved in the pathogenesis of essential hypertension [9].

ROS are mainly produced by mitochondria. There are many processes that generate superoxide as a natural byproduct of mitochondrial metabolism, including leakage from electron transport chain (ETC.) enzymes during oxidative phosphorylation and the tricarboxylic acid (TCA) cycle. In normal physiological conditions, ROS participate in critical signaling pathways to mediate adaptive responses and regulate diverse biologies, including cell growth and differentiation [10]. However, once the balance between production and detoxification pathways is broken, excess ROS generation, it will lead to accumulated oxidative damage of critical macromolecules, such as DNA, RNA, proteins and lipids [11]. Oxidative stress (OS) contributes to vascular injury by promoting inflammation, endothelial dysfunction and increased vascular tone, leading to altered vascular contractility, structural remodeling, and hypertension as well as other forms of cardiovascular disease [12].

SIRT3 is one of the seven mammalian sirtuins, which are a conserved family of proteins possessing NAD^+^-dependent deacetylase activity. Of the seven sirtuins, SIRT3 is the only sirtuin analogue whose increased expression was shown to be associated with longevity of humans [13, 14]. SIRT3 is a protein of tremendous potential, which can modulate avariety of cellular processes, including growth arrest, apoptosis, senescence, and metabolism. SIRT3 resides primarily in the mitochondria and has been shown to bind and deacetylate several metabolic and respiratory enzymes that regulate important mitochondrial functions [15]. Studies in the past few years have shown that SIRT3 regulate the production and clearance of ROS through deacetylation of numerous mitochondrial enzymes [16–22]. For instance, SIRT3 can reduce ROS production via activation of long chain fatty acyl-CoA dehydrogenase (LCAD), succinate dehydrogenase (SDH) and NADH dehydrogenase, as well as improve ROS clearance through activation manganese superoxide dismutase (MnSOD) and catalase (CAT). In the heart, SIRT3 has been found to block development of cardiac hypertrophy and protect cardiomyocytes from oxidative stress-mediated cell death [20].

In this study, we investigated the influence of AngII on the protein expression of SIRT3 in human umbilical vein endothelial cells (HUVECs) and the role of SIRT3 in AngII-induced HUVECs dysfunction.

## Methods

### Cell culture, treatment and transfection

Normal Primary Human umbilical vein endothelial cells (ATCC® PCS-100-010™) were maintained in endothelial cell medium (ECM; Sciencell, USA) in humidified incubator with 5 % CO_2_ and 95 % air at 37 °C until desired cell density was reached. Confluent cells cultured up to 3–7 passages were first starved in 0.5 % fetal bovine serum ECM for 24 h before exposure to AngII (Sigma; 10^−7^, 10^−6^, 10^−5^ mol/L) for another 24 h.

The SIRT3 siRNA used was synthesized by Shanghai GenePharma (Shanghai). Negative control siRNA, which does not target any known gene, was used as a negative control. Cells were transfected with the siRNAs by the Lipofectamine 2000 (Invitrogen) according to the manufacturer’s instructions and examined by western blot analysis. Cells transfected with SIRT3 siRNA were then treatment with or without AngII (10^−6^ mol/L) for 24 h.

### Assessment of intracellular ROS levels

Intracellular ROS was detected with 2′,7′-dichlorodihydro-fluorescein diacetate (DCFH-DA; Sigma). Treated cells were stained with 10 μM DCFH-DA and incubated in dark at 37 °C for 20 min. The stained cells were washed 3 times with PBS, and then detected under a confocal laser scanning microscope (ZEISS LSM 710, Germany) at an excitation wavelength of 488 nm and an emission wavelength of 520 nm.

### Western blot analysis

Total protein was extracted by total protein extraction kit (Beyotime, Jiangsu, China). Protein concentration was measured by BCA protein assay kit (Beyotime, Jiangsu, China). Each protein sample was loaded onto a 12 % SDS-PAGE, and then transferred to polyvinylidene difluoride membranes (Millipore, Billerica, MA). The membrane was blocked with 5 % milk (in 40 mL TBST) for 2 h before incubated with primary antibodies against β-actin, SIRT3 and endothelial nitric oxide synthase (eNOS) (all Cell Signaling Technology) at 4 °C overnight. After being washed with TBST for 3 times, the membranes were incubated with secondary anti-rabbit horseradish peroxidase-labeled antibody for 2 h and visualized by enhanced chemiluminescence (Millipore, Billerica, MA).

### Real-time PCR

Total RNA was isolated using TRIzol (Invitrogen, Carlsbad, CA, USA). The first-strand cDNAs were synthesized from 2 μg of total RNAs in 20 ml reaction using Prime Script RT reagent kit (TaKaRa Biotechnology, Dalian, China). Quantitative PCR was performed in Eppendorf Mastercycler ep realplex detection system (Eppendorf, Hamburg, Germany) by Fast start universal SYBR green master (Roche, Germany) according to the manufacturer’s protocol. The relative mRNA levels were as normalized to GAPDH and calculated as 2^−ΔΔCT^.

The primers for PCR are as follow: SIRT3, forward 5′- CCCCAAGCCCTTTTTCACTTT-3′ and reverse 5′-CG ACACTCTCTC AAGCCCA-3′; GAPDH, forward 5′-GAGTCAACGGATTTGGTCG T-3′ and reverse 5′-AATGAAGGGGTCATTGATGG-3′.

### eNOS activity assay

eNOS activity was measured by use of a nitricoxide synthase assay kit (Beyotime, Jiangsu, China). Treated cells in 96-well plates were incubated with 100 μL NOS assay buffer (plus iNOS inhibitor) and 100 μL reaction buffer (containing 3-amino,4-aminomethayl- 2′,7′-difluorescein diacetate, DAF-FM DA) per well for 60 min at 37 °C. DAF-FM reacts with NO free radicals, becoming fluorescent in the presence of NO. Then the fluorescent identity was determined by microplate reader (INFINITE M200, Switzerland) analysis at excitation 495 nm and emission 515 nm.

### Measurement of NO concentration

The NO levels in the cell culture supernatant were measured by using nitric oxide assay kit (Beyotime, Jiangsu, China) in nitrate reductase method. Briefly, standards were dilute to 0, 1, 2, 5, 10, 20, 40, 60, 100 μM/L with ECM. 50 μL standards or samples were mixed with 50 μL Griess Reagent I and 50 μL Griess Reagent II at room temperature, and the absorbance at 540 nm was measured using a microplate reader (INFINITE M200, Switzerland). The concentration of NO in the samples was calculated by using a standard curve of sodium nitrite.

### Statistical analysis

The data are expressed as mean ± SEM The difference between groups was analyzed by a one-way analysis of variance. *P* < 0.05 was considered statistically significant.

## Results

### Dose-dependent effects of AngII on SIRT3 expression of HUVECS

**Fig. 1:**
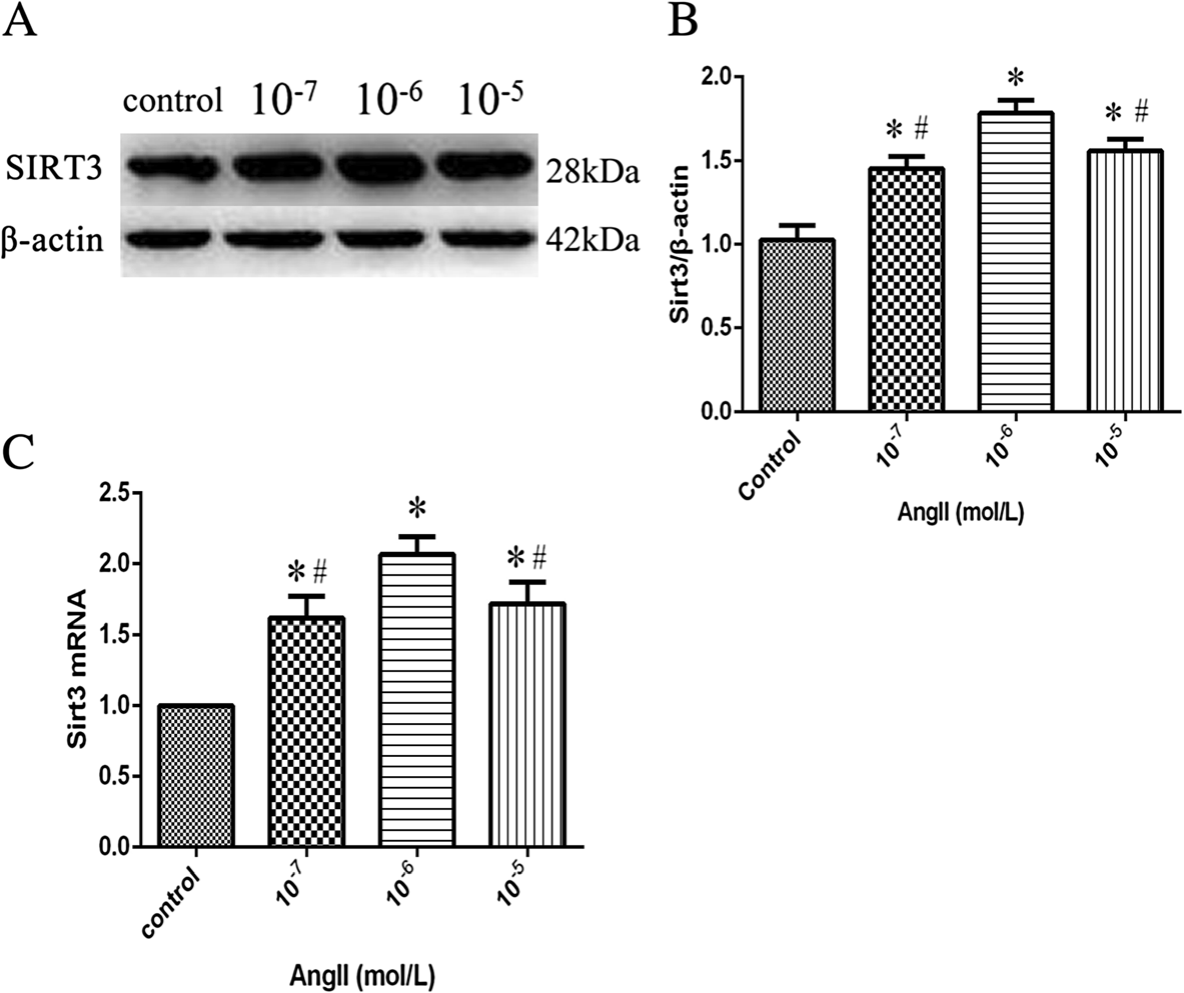
Dose-dependent effects of AngII on SIRT3 expression. Cells were stimulated by AngII of 0, 10^−7^,10^−6^,10^−5^ mol/L for 24 h, SIRT3 protein expression were determined by western blot in each group (**a**) and Quantification of SIRT3 in each group, β-actin as a reference (**b**). SIRT3 mRNA expression were determined by RT-PCR (**c**). Data are the mean ± SEM from three separate experiments. ^*^ *P* < 0.05 vs. control, ^#^ *P* < 0.05 vs.10^−6^ mol/L group

Treatment with 0, 10^−7^, 10^−6^, 10^−5^ mol/L AngII for 24 h resulted in a significant increase of SIRT3 protein expression in HUVECS, with the peak at 10^−6^ mol/L (Fig. 1a and b). The mRNA expression levels of SIRT3 were also increased significantly (Fig. 1c). The dose of 10^−6^ mol/L was selected for the treatment condition in subsequent experiments.

### Screening of SIRT3 siRNA sequences

**Fig. 2:**
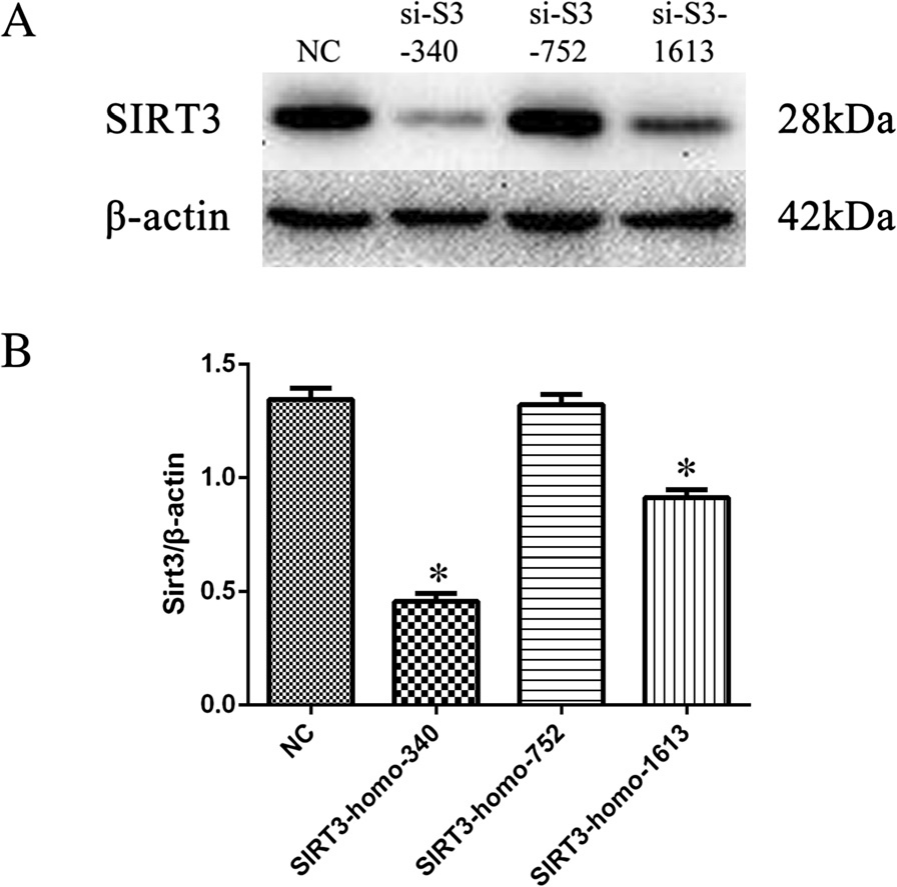
Screening of SIRT3 siRNA sequences. Cells were transfected by SIRT3 siRNA of different sequences. SIRT3 expression were determined by western blot in each group (**a**) and Quantification of SIRT3 in each group, β-actin as a reference (**b**). Data are the mean ± SEM from three separate experiments. ^*^ *P* < 0.05 vs. Negative Control (NC)

Inhibition efficiency of SIRT3 siRNA sequences were examined by western blot analysis. The inhibition efficiency of SIRT3-homo-340 is the highest (Fig. 2a and b). SIRT3 siRNA sequences SIRT3-homo-340 was screened for subsequent experiments.

### Knockdown of SIRT3 worsen the AngII-induced effects on HUVECS

**Fig. 3:**
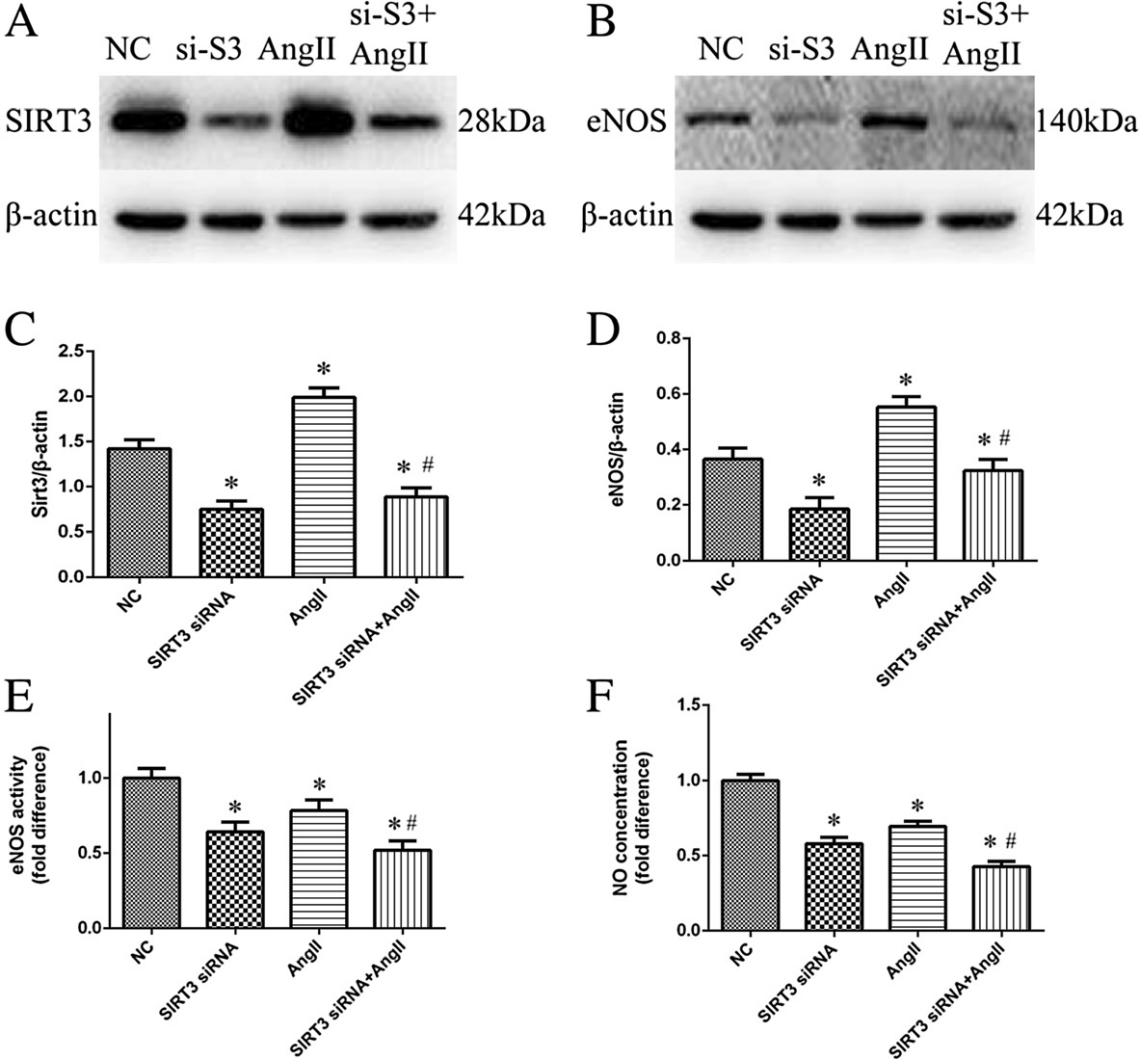
Knockdown of SIRT3 worsen the AngII-induced effects on HUVECS. Cells transfected with negative control siRNA or SIRT3 siRNA were then treatment with AngII for 24 h. SIRT3 expression were determined by western blot in each group (**a**) and Quantification of SIRT3 in each group, β-actin as a reference (**c**). eNOS expression were determined by western blot in each group (**b**) and Quantification of eNOS in each group, β-actin as a reference (**d**). eNOS activity in each group (**e**), NO concentration in each group (**f**). Data are the mean ± SEM from three separate experiments. ^*^ *P* < 0.05 vs. Negative Control (NC), ^#^ *P* < 0.05 vs. AngII group

AngII (10^−6^ mol/L) induced an obvious pathological phenotype of HUVECS, as shown by a reduced eNOS activity (0.78, ^*^P < 0.05 versus NC, Fig. 3e) and reduced NO concentration (0.70,^*^P < 0.05 versus NC, Fig. 3f), however, eNOS expression increased in response to AngIIstimuli (Fig. 3b and d). When SIRT3 was knocked down using siRNA, the activity of eNOS in SIRT3 siRNA + AngII group decreased more significantly than that in AngII group (0.52 in SIRT3 siRNA + AngII group, 0.78 in AngII group, ^#^*P* < 0.05, Fig. 3e). The concentration of NO further decreased when pretreated with SIRT3 siRNA (0.43 in SIRT3 siRNA + AngII group, 0.70 in AngII group, ^#^*P* < 0.05, Fig. 3f). In addition, treatment with SIRT3 siRNA reduced eNOS expression under conditions with or without AngII (Fig. 3b and d).

### The effect of SIRT3 on ROS level in HUVECs treated with AngII

**Fig. 4:**
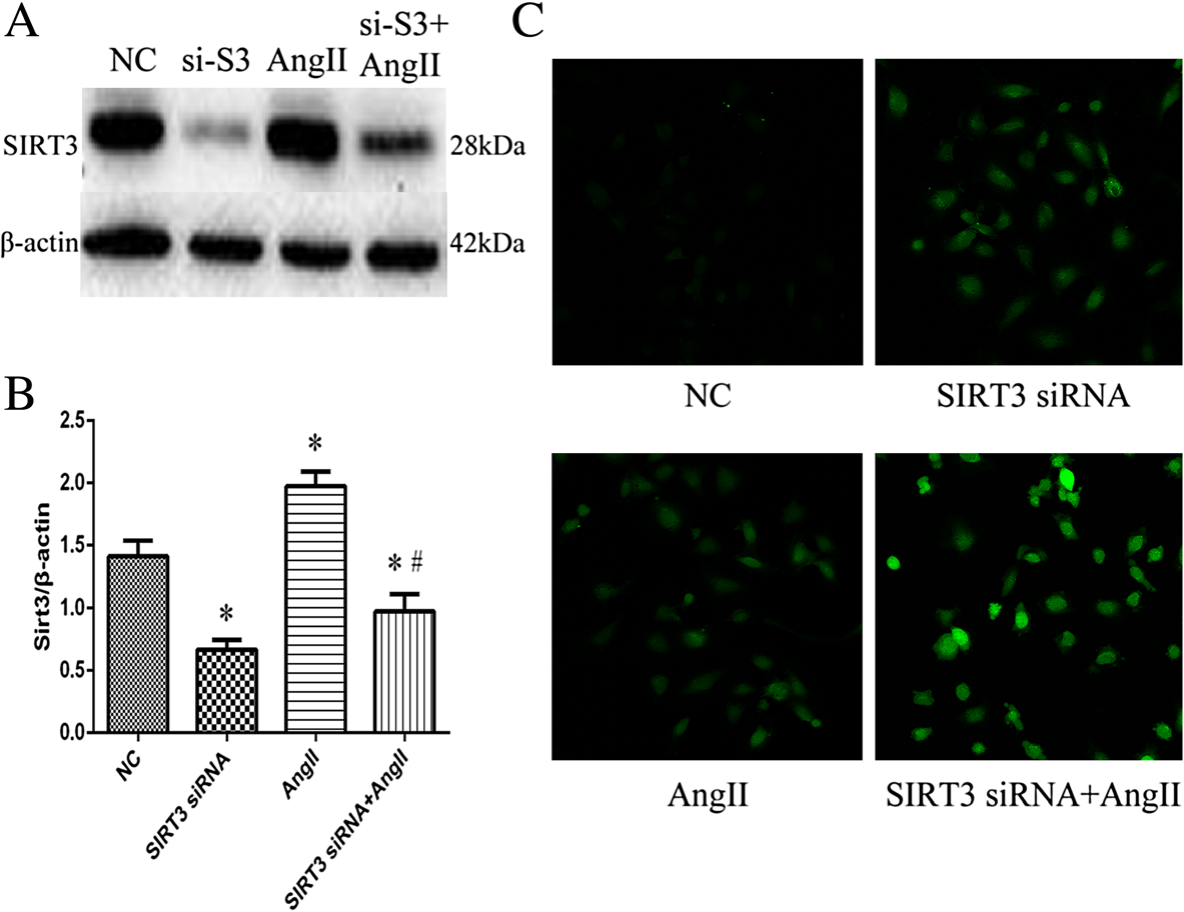
The effect of SIRT3 on ROS level in HUVECs treated with AngIIThe effect of SIRT3 on ROS level in HUVECs treated with AngII. Cells pretreated with negative control siRNA or SIRT3 siRNA were exposed to AngII for 24 h. SIRT3 expression were determined by western blot in each group (**a**) and Quantification of SIRT3 in each group, β-actin as a reference (**b**). ROS levels in each group (**c**). Original magnification × 200. Data are the mean ± SEM from three separate experiments. ^*^ *P* < 0.05 vs. Negative Control (NC), ^#^ *P* < 0.05 vs. AngII group

To confirm the role of SIRT3 in AngII-induced oxidative stress injury in HUVECs, cells pretreated with negative control siRNA or SIRT3 siRNA were exposed to AngII for 24 h. SIRT3 significantly increased ROS level of cells with or without intervention of AngII (Fig. 4c).

## Discussion

In this study, we found that AngII could upregulate SIRT3 expression in HUVECs distinctly. SIRT3 siRNA could worsen AngII induced endothelial dysfunction via increase generation of ROS.

Endothelial dysfunction has been implicated in the pathophysiology of atherosclerosis, hypertension, and heart failure. It could be defined as impairment characterized of reduced vasodilation, a proinflammatory state, and prothrombotic setting. Oxidatative stress has been confirmed as one of the major mechanisms responsible for the formation and development of endothelial dysfunction [6]. There is considerable evidence supporting the view that oxidative stress is involved in the pathophysiology of cardiovascular diseases. Oxidative stress can result not only from increased ROS production, but also from decreased ROS scavenging ability. Hypertension is reported to be associated with decreased antioxidant systems as well as enhanced production of ROS [23]. NOX is the primary biochemical source of ROS in the vasculature, particularly of superoxide. The vasculature is rich sources of NOX-derived ROS, which under pathological conditions play an important role in vascular damage [24]. AngII is the most studied stimulus of NOX activation. Previous studies indicated that in rats and mice made hypertensive by AngII infusion, expression of NOX subunits, oxidase activity, and generation of ROS are all increased [25]. Likely the most well-known function of NOX derived superoxide is inactivation of NO to form peroxynitrite, leading to impaired endothelium-dependent vasodilation and uncoupling of eNOS to produce additional superoxide [26]. In the vasculature, NOX activation has been strongly associated with hypertension. The primary function of eNOS is NO production. NO is known as a key paracrine regulator of vascular tone [27]. Physiologically, NO inhibits leukocyte-endothelial cell adhesion, VSMC proliferation and migration, and platelet aggregation to maintain the health of the vascular endothelium. The reduction in bioavailability of NO in the vasculature decreases vasodilatory capacity and contributes to endothelial dysfunction. Our results confirmed these findings that AngII treatment could increase generation of ROS and decrease eNOS activity and total level of NO, while eNOS protein expression was up-regulated. We consider that AngII induced up-regulated of eNOS protein expression may be a compensatory reflection.

Mitochondria are the primary sources of ROS, as there are many processes that generate superoxide as a natural byproduct of mitochondrial metabolism, such as leakage from ETC. enzymes during oxidative phosphorylation and the TCA cycle. SIRT3 resides primarily in the mitochondria and has been shown to regulate production and clearance of ROS via deacetylation of numerous mitochondrial enzymes. Recently researches have reported that SIRT3 prevented cardiac hypertrophic response by scavenging ROS through activation of Foxo3a and upregulation of MnSOD and CAT [20]. In our study, SIRT3-siRNA increased HUVECs ROS under the stimulation of AngII. This is consistent with a previous paper showing that SIRT3 is required to reduce cellular ROS in particular under the conditions of stress [28].

NAD^+^ is a substrate for the class III NAD^+^-dependent deacetylase Sirtuins, and a powerful activator of the Sirtuin activity. The enzymatic activity SIRT3 is sensitive to NAD^+^/NADH ratio [29]. It has been proved that NADH accumulation and decreased NAD^+^/NADH ratio caused by complex I deficiency inhibits SIRT3 activity [30]. The mechanism of SIRT3 expression increase induced by AngII can be presumed that a change in the cellular NAD^+^/NADH ratio, in part, contribute to increased levels of SIRT3, based on studies with other sirtuins, It has been reported that decreased NADH levels following calorie restriction, which increased the NAD^+^/NADH ratio, elevated levels of yeast Sir2 [31]. In addition, research shows that SIRT3 expression is regulated in response to mitochondrial oxidative stress in primary hippocampal cell [32]. Therefore, we speculate that the upregulation of SIRT3 expression induced by AngII could be due to the increase of NAD^+^/NADH ratio and accumulation of ROS through activation of NOX. We considered that the upregulation of SIRT3 induced by AngII was a protective effect as compensative response. In our study, we use SIRT3 siRNA to knock down SIRT3 expression in HUVECs, and knock down of SIRT3 worsen AngII-induced pathological phenotype of HUVECS as shown by reduced eNOS activity, NO concentration and eNOS expression.

## Conclusion

These data suggest that SIRT3 plays a protective role in AngII-induced HUVECs dysfunction via regulation of ROS generation. Our study provides evidences for further researches on the role of SIRT3 in cardiovascular diseases.

## Abbreviations

AngII: Angiotensin II
CAT: Catalase
eNOS: Endothelial nitric oxide synthase
ETC.: Electron transport chain
HUVECs: Human umbilical vein endothelial cells
LCAD: Long chain fatty acyl-CoA dehydrogenase
NOX: Nicotinamide adenine dinucleotide phosphate oxidase
OS: Oxidative stress
ROS: Reactive oxygen species
RAAS: Renin-angiotensin-aldosterone system
SDH: Succinate dehydrogenase
TCA: Tricarboxylic acid
